# Community-Level Mental Health and Psychosocial Support During Armed Conflict: A Cohort Study From the Democratic Republic of the Congo, Mali, and Nigeria

**DOI:** 10.3389/fpubh.2022.815222

**Published:** 2022-03-28

**Authors:** Ida Andersen, Rodolfo Rossi, Ives Hubloue

**Affiliations:** ^1^Health Unit, International Committee of the Red Cross, Geneva, Switzerland; ^2^Research Group on Emergency and Disaster Medicine, Vrije Universiteit Brussel, Brussels, Belgium

**Keywords:** ICRC, MHPSS, Africa, conflict, sexual violence, red cross red crescent movement

## Abstract

**Introduction:**

Community-level mental health and psychosocial support (MHPSS) was the first type of MHPSS program launched by the International Committee of the Red Cross (ICRC) back in 2004. Standardized beneficiary-level monitoring was put in place in late 2018. This is the first study to explore whether this type of program correlates, as intended, with reduced psychological distress and increased daily functioning.

**Methods:**

Between December 2018 and June 2020, 6,413 victims of violence received MHPSS through 32 community-level projects in the Democratic Republic of the Congo (DRC), Mali and Nigeria. Symptoms of psychological distress (IES-R or DASS21) and daily functioning (ICRC scale) were assessed before and after the intervention and logistical regression models were used to identify predictors of these symptoms.

**Findings:**

Victims of the violence committed by weapon bearers were more likely to show high levels of anxiety prior to MHPSS (aOR 3.51; *p* < 0.0001). Also, victims of physical violence were more likely to show high levels of stress (aOR 1.49; *p* < 0.0001), whereas victims who had witnessed physical violence were more like to report high levels of depression (aOR 2.54; *p* < 0.0001). The most common perpetrators were weapon bearers (76%) and the most common type of violence was rape (46%). Lack of social support stood out as a predictor of both high anxiety (aOR 2.10; *p* < 0.0001) and post-traumatic stress (aOR 2.04; *p* < 0.0001) prior to MHPSS. Following MHPSS, the vast majority of beneficiaries reported a reduction in distress on the DASS21 (96.58%) and the IES-R scales (92.70%) as well as an increase of functioning (82.26%). Adherence to group therapy (seven sessions on average) was stronger than adherence to individual therapy (four sessions on average). A linear trend was found between length of treatment and likelihood of reporting reduced symptoms of depression. Having suffered destruction or loss of property or income predicted less improvement of functioning following MHPSS (aOR 0.90; *p* = 0.044).

**Conclusion:**

Receiving community-level MHPSS is associated with increased wellbeing among the vast majority of beneficiaries. To further enhance the intended health outcomes, it is recommended to increase the length of treatment per beneficiary (30 days minimum) and address, where relevant, the financial consequences of violence. Also, a longitudinal study is recommended to assess longer-term changes in MHPSS symptoms.

## Introduction

### Community-Level MHPSS in the ICRC

For more than 150 years, the International Committee of the Red Cross (ICRC) has offered humanitarian protection and assistance to victims of armed conflict and other situations of violence. Mental health and psychosocial support (MHPSS) is one of the most recent additions to the wide-ranging assistance programs of the ICRC (1) and aims to address the specific MHPSS consequence of violence, including sexual violence.

Community-level MHPSS for victims of violence was the first type of MHPSS program launched by the ICRC. Starting with the *maisons d'écoute* counseling centers in the Democratic Republic of the Congo (DRC) in 2004 (2), similar types of projects are now running in 15 countries worldwide, reaching more than 32,000 direct beneficiaries in 2020 alone.

Community-level MHPSS programs are implemented in collaboration with a local partner such as a local association, non-government organization or the Red Cross Red Crescent National Society. These programs are part of a larger effort to holistically address the needs of victims of violence, which can include health (3), economic security, water and habitat, protection and prevention.

### From Monitoring Outputs to Conducting Operational Research

Initially, the monitoring of ICRC MHPSS programs was focused on *outputs* such as “number of training sessions organized.” In 2008, the ICRC introduced the results-based management approach (4), which shifted focus to the *outcomes* of each humanitarian project, such as “percentage of participants who improved their knowledge” following training.

The “Framework for monitoring and evaluation of MHPSS programs in emergency settings” (5), published by the Inter-Agency Standing Committee (IASC) in 2017, intensified efforts at a global level to improve and harmonize MHPSS monitoring and evaluation tools. Within the International Red Cross and Red Crescent Movement (“Movement”) (6), the International Federation of Red Cross and Red Crescent Societies (IFRC) published a “Monitoring and Evaluation Framework for Psychosocial Support Interventions” (7) in 2017, targeting National Red Cross and Red Crescent Societies.

Similarly, in 2018, the ICRC MHPSS team adopted an electronic case management system with standardized monitoring tools, such as intake forms and psychometric scales, that made it possible to track the evolution of each direct beneficiary before, during and after MHPSS care.

These initiatives lay the foundation for the Movement “Policy on Addressing Mental Health and Psychosocial Needs” (8) adopted during the 33rd International Conference^1^ in December 2019. One of the policy's eight statements is devoted to ensuring that MHPSS programs are “informed by evidence,” “ensure quality of care” and that the Movement “contributes, where possible, to data collection [and] research.”

The present study would not have been possible without this immense work of improving and harmonizing ICRC MHPSS monitoring tools. It aims to walk the talk in terms of carrying out operational research to inform practice and contribute to the evidence base for MHPSS programs in conflict settings.

### The MHPSS Pyramid of the International Red Cross and Red Crescent Movement

**Figure 1:**
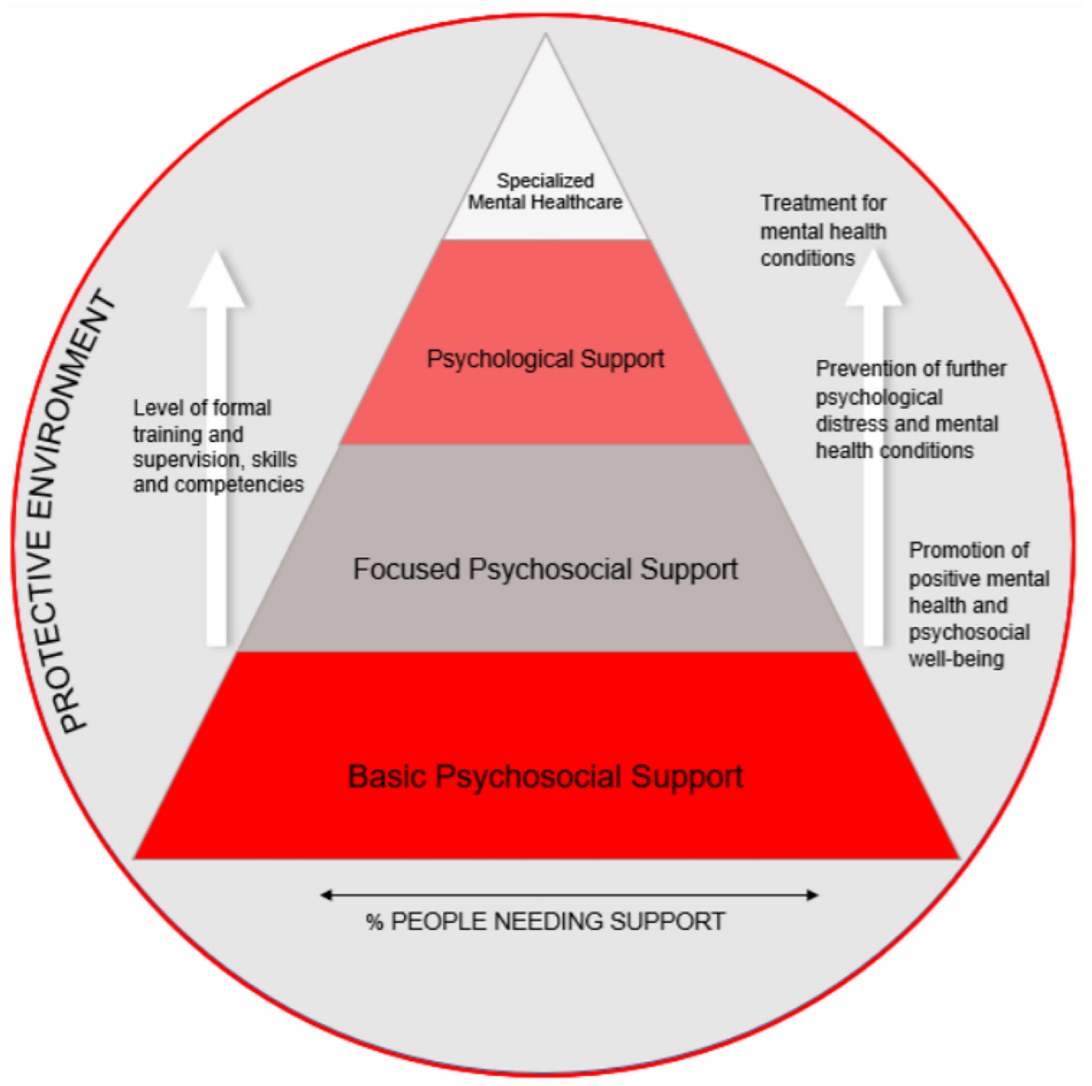
MHPSS Movement pyramid.

While the IASC MHPSS pyramid (5) is intended for emergency settings only, the MHPSS Movement pyramid is intended for all contexts in which the Movement works. It contains four different layers (Figure 1) (8).

First, *basic psychosocial suppor*t that targets 100% of a population affected by violence and seeks to promote resilience by raising awareness, for example, on common MHPSS consequences of exposure to violence, including sexual violence and locally available services. In the DRC, Mali and Nigeria, partner organizations such as local associations or the National Red Cross Society are trained and supervised by ICRC MHPSS teams to carry out awareness-raising activities in selected areas on themes of relevance to the context.

Second, *focused psychosocial support* aims to prevent the development of more severe mental health consequences of exposure to violence. In Nigeria, the ICRC MHPSS teams train and supervise National Society volunteers to conduct group sessions for victims of violence that include a peer support component in which the participants learn from one another.

Third, *psychological support* is the predominant layer in ICRC MHPSS programs for victims of violence at community level. It addresses the needs of victims of violence that present more severe psychological distress and involves individualized counseling and/or group interventions. In the DRC and Mali, the ICRC MHPSS teams train and supervise partner organizations such as local associations or the National Red Cross Society to assess the unique difficulties of each beneficiary in terms of psychological distress and daily functioning using psychometric tools. A treatment plan is laid down, and the beneficiary is offered individual counseling sessions. In Nigeria, the group sessions offered in collaboration with the Nigerian Red Cross also target the psychological needs of victims of violence and use similar psychometric monitoring tools.

Fourth, *specialized mental health care* involves highly advanced psychological and psychiatric services. In the DRC, Mali and Nigeria, the ICRC does not offer these services directly but aims to refer victims of violence for whom the three lower levels of MHPSS care do not suffice to specialized services available through the Ministries of Health or other actors.

### Research Question

In this study, we look specifically at the *psychological support layer* of community-level MHPSS programs by examining the data available from the ICRC's clinical work with victims of violence at community level in the DRC, Mali and Nigeria. The aim is two-fold—first, to identify predictors of high psychological distress and low functioning prior to psychological support and, second, to identify predictors of reduced distress and increased functioning following psychological support.

## Methods

### Study Design

We conducted a non-controlled retrospective cohort study of 6,413 victims of violence who received psychological support between December 2018 and June 2020 through 32 community projects in the DRC, Mali and Nigeria. The data were collected routinely for clinical follow-up and internal monitoring purposes.

### Target Population

The target population of ICRC community-level MHPSS programs consisted of civilian men, women, boys and girls directly affected by armed conflict or other situations of violence. According to the ICRC's mandate, these programs were set up in areas where violence is committed mainly by weapon bearers. Thus, they targeted victims of a form of violence that could be considered a breach of IHL, i.e., the principle of distinction that prohibits indiscriminate attacks affecting the civilian population (9).

Psychological support was offered to victims that presented particularly high levels of psychological distress and low functioning as a result of exposure to conflict-related violence.

### The Intervention

#### Pre-assessment

**Figure 2:**
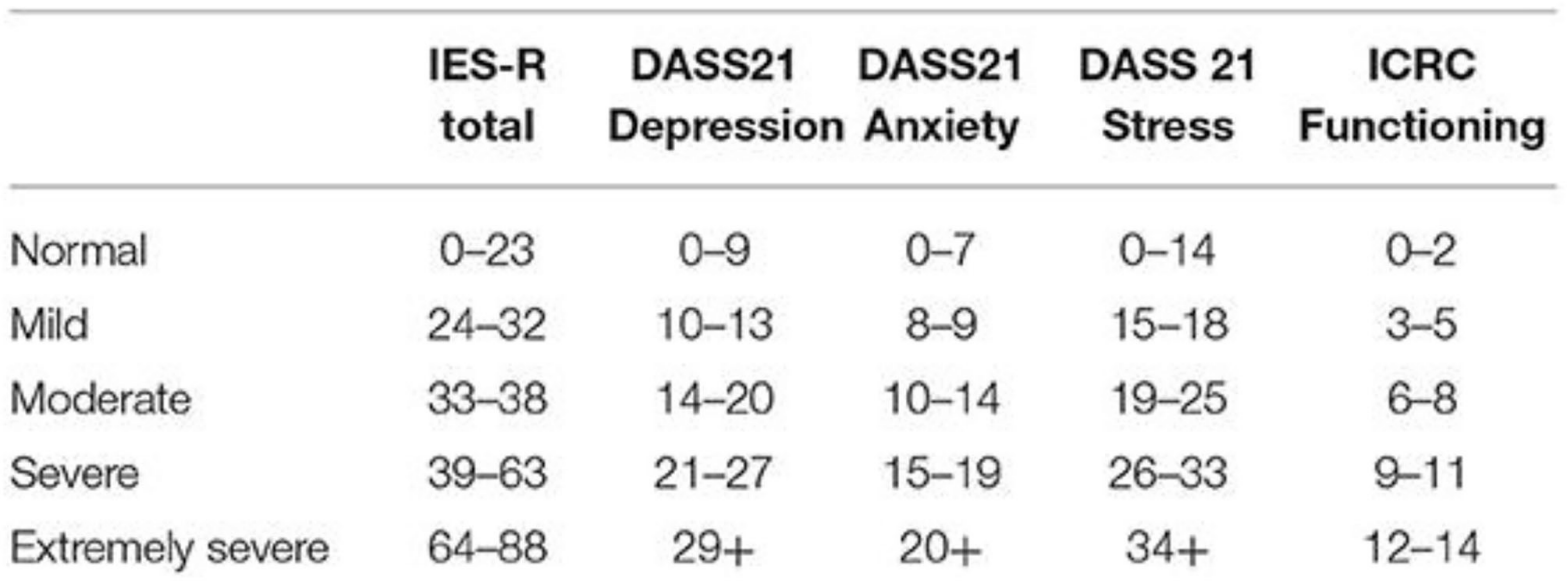
Categorization of IES-R, DASS 21, and ICRC functioning scores used in this study.

To evaluate the pertinence of offering psychological support and prepare an individualized treatment plan, levels of psychological distress were assessed using either the Depression, Anxiety and Stress Scale with 21 items (DASS21) or the Impact of Events Scale Revised (IES-R). In addition, the ICRC functioning scale was used to estimate the level of daily functioning of each beneficiary (10) (Figure 2).

#### Individual Psychological Support

Victims of violence in the DRC and Mali received individual psychological support by lay counselors called *agents psychosociaux* (APS) who were trained and supervised by the ICRC MHPSS team. As described in a previous publication (11), a short-term solution-oriented approach (11, 12) was adopted to empower the beneficiary to reflect upon and resolve his or her specific problems. In addition to offering psychological support, referrals to local service providers were made according to needs and availability. Some counseling centers could also briefly accommodate victims of violence who lived far from the counseling center, who faced family disputes, stigma or other difficulties that prevented them from returning to their homes.

#### Group Psychological Support

Victims of violence in Nigeria received group psychological support by ICRC-trained lay counselors working for the Nigerian Red Cross Society (NRCS). After one or several individual preparatory sessions that allowed the counselors to get to know the beneficiaries and group them according to their profiles, 10 group sessions were organized, focusing on the following themes:

I. Introduction
II. Loss and grief
III. Flashbacks and intrusive memories
IV. Sleep and nightmares
V. Anger and irritability
VI. Psychosomatic pain
VII. Guilt and self-blame
VIII. Family problems
IX. Summary
X. Closure an post-evaluations

Each session included a strong psychoeducational component and introduced the participants to adaptive coping skills.

#### Post-assessments

Following the psychological support, levels of psychological distress and daily functioning were assessed once again, using the same psychometric tools as during the pre-assessment phase.

### Dataset

The following variables from the dataset were used in the study:

#### Demographic Information

Country, gender, age, civil status, resident/migrant/internally displaced, education level, occupation, and number of children.

#### Type of Violence Experienced

Victim of physical violence (excluding rape, attempted rape, and torture), witness of physical violence, rape, attempted rape, incest, forced marriage, forced prostitution, victim of trafficking/smuggling, kidnapping/hostage taking including sexual violence, kidnapping/hostage taking excluding sexual violence, killing of a loved one, disappearance of a loved one, forced recruitment, torture/ill-treatment, insults/threats, others.

#### Place of Violence

Home, school/work, on the road/while going somewhere, during combat, while fleeing or in a camp for internally displaced people.

#### Alleged Perpetrator

Type (partner, family member, known civilian, unknown civilian, military or armed group) and number of perpetrators.

#### Vulnerability Factors

Destroyed/lost property and/or income, mother head of household, natural death of loved 1 < 2 years ago, natural death of loved 1 more than 2 years ago, parents missing, caretaker neglect, severe or chronic medical/physical condition, severe or chronic mental health condition, suffering from stigma due to an illness, congenital anomaly, marginalization and social discrimination, absence of social support network and forced to flee.

#### The MHPSS

Timing (number of days between latest violence and first consultation), number of individual sessions received, number of group sessions received and length of support (number of days between pre- and post-assessment).

#### Pre- and Post-assessments

Levels of psychological distress were measured through the Depression, Anxiety and Stress Scale with 21 items (DASS21) in DRC and Mali, and through the Impact of Event Scale Revised (IES-R) in Nigeria. Daily functioning was measured through the ICRC functionality scale in all countries.

### Data Management and Statistical Analysis

All categorical data were numerically coded. Quantitative/continuous variables (i.e., pre- and post-scores) were either kept as such or categorized depending on the type of analysis. Categorization of continuous variables was done either by identifying the median to divide the study participants in two even-sized groups or by using established clinical cut-offs (see Section Dataset).

The dataset was created in Microsoft Excel with two independent data clerks to control for potential typing mistakes. The electronic dataset was protected by a password, which was changed every 3 months. The dataset was transferred to STATA™, version MP 16.0 for analysis.

All quantitative variables were explored by defining their means (and standard deviation), medians and quartiles. Comparisons of means were tested through the *t*-test, and the corresponding *p*-value was reported; 95% confidence intervals (95% CI) were calculated around means and means differences. Categorical variables were explored through percentages and tested using the Chi^2^-test to retrieve the corresponding *p*-value; 95% CIs were calculated around these percentages.

To measure associations between pre- and post-scores and the other variables (crude and multivariable), logistic regression models were fitted to calculate odds ratios (OR) with corresponding 95% CIs and *p*-values from the Wald test. All variables were initially explored in a crude model and included in a multivariable model and presented only if statistically significant. We considered as significant *p*-values < 0.05.

## Findings

### The Study Population

Of the 6,413 victims of violence included in the study, 81% (*N* = 6,413) were from the DRC, 3% from Mali and 16% from Nigeria. As many as 80% were female and the mean age was 32 (Table 1). The main types of violence experienced by the patients were rape (46%) and physical violence (42%). The vast majority of the alleged perpetrators weapon-bearers (76%). The most common place of violence was on the road (39%) and in the victim's home (34%). Additional vulnerability factors mentioned by the patient during the first session included destruction or loss of property or income (60%) and being a mother head of the household (30%).

**Table 1:** Characteristics of the study population and the MHPSS.

	***n***	**%**
**Country (*****N*** **=** **6,413)**		
Democratic Republic of the Congo	5,190	80.93
Mali	184	2.87
Nigeria	1,039	16.20
**Gender (*****N*** **=** **6,287)**		
Male	1,238	19.69
Female	5,049	80.31
**Age (*****N*** **=** **6,345)**		
0–17	550	8.67
18–24	1,178	18.57
25–34	2,172	34.23
35–44	1,450	22.85
45–81	995	15.68
**Civil status (*****N*** **=** **6,220)**		
Single (incl. children)	1,401	22.52
Married	3,601	57.89
Partner abroad	22	0.35
Partner missing	72	1.16
Divorced/Separated	350	5.63
Widow/er	721	11.59
Other	53	0.85
**Resident-migrant/IDP status (*****N*** **=** **6,104)**		
Resident	4,437	72.69
Internally displaced	1,633	27.31
**Education level (*****N*** **=** **6,190)**		
Illiterate	2,023	32.68
Primary	2,402	38.80
Secondary	1,668	26.95
High	97	1.57
**Occupation (6,412)**		
Unemployed	134	2.09
Student	624	9.73
Farming/cattle	3,160	49.28
Shop/business	1,210	18.87
Other jobs	1,284	20.02
**Number of children (*****N*** **=** **5,514)**		
0	879	15.94
1	305	5.53
2	516	9.36
3	659	11.95
4	723	13.11
5	700	12.69
6	632	11.46
7–20	1,100	19.95
**Type of violence experienced (*****N*** **=** **6,413)**		
Victim of physical violence excluding rape, attempted rape and torture	2,718	42.38
Witness of physical violence	1,352	21.08
Rape	3,937	45.80
Attempted rape	604	10.82
Incest	262	4.09
Forced marriage	571	8.90
Forced prostitution	24	0.37
Victim of trafficking/smuggling	28	0.44
Kidnapping/hostage taking including sexual violence	144	2.25
Kidnapping/hostage taking excluding sexual violence	304	4.74
Killing of a loved one	696	10.85
Disappearance of a loved one	356	5.55
Forced recruitment	64	1.00
Torture/Ill-treatment	301	4.69
Insults/threats	246	3.85
Other	447	6.97
**Alleged perpetrator (*****N*** **=** **6,011)**		
Partner	73	1.21
Family member	193	3.21
Known civilian (non-family member)	508	8.45
Unknown civilian	616	10.25
Weapon-bearers	4,564	75.93
No information	57	0.95
**Other factors of vulnerability highlighted by the patient during the first session (not mutually exclusive) (*****N*** **=** **6,413)**		
Destroyed/lost property and/or income	3,879	60.49
Mother head of household	1,900	29.63
Natural death of loved 1 <2 years ago	776	12.10
Natural death of loved 1 more than 2 years ago	597	9.31
Parents missing	575	8.97
Caretaker neglect	163	2.54
Severe or chronic medical/physical condition	414	6.46
Severe or chronic mental health condition	1,433	22.35
Suffering from stigma due to an illness	26	0.41
Congenital anomaly	62	0.97
Marginalization and social discrimination	146	2.28
Absence of social support network	431	6.72
Forced to flee	206	3.21
**Number of perpetrators (*****N*** **=** **5,598)**		
None	34	0.61
One	1,645	29.39
Several	3,919	70.01
**Place of violence (*****N*** **=** **6,025)**		
Home	2,037	33.81
School/work	897	14.89
On the road/while going somewhere	2,323	38.56
During combat	145	2.41
While fleeing	194	3.22
In an IDP camp	10	0.17
Other	410	6.95
**Timing: days between latest violence and first consultation (*****N*** **=** **3,322)**		
0–2	361	10.87
3–14	991	29.83
15–90	770	23.18
91–365	285	8.58
<365	915	27.54
**Number of individual sessions excluding pre- and post-assessments (*****N*** **=** **1,319)**		
1–2	95	7.20
3–4	741	56.18
5–6	460	34.87
7–10	23	1.74
**Number of group sessions excluding pre- and post-assessments (*****N*** **=** **691)**		
1–2	82	11.87
3–4	82	11.87
5–6	154	22.29
7–12	373	53.98
**Length of MHPSS: days between pre- and post-assessment (*****N*** **=** **2,268)**		
<8	95	4.19
8–14	135	5.95
15–30	561	24.74
31–60	516	22.75
61–90	316	13.93
91–120	343	15.12
121–150	180	7.94
>150	122	5.38

### The MHPSS

The timing of the MHPSS was most commonly 3–14 days following exposure to violence (30%). The most common length of the support was 15–30 days (25%) and 31–60 days (23%). In addition to the first and last sessions consisting of pre- and post-assessments, patients most commonly received 3–4 individual follow-up session (56%). Patients receiving group support attended an average of seven sessions.

### Levels of Distress and Functioning Before and After MHPSS

Prior to receiving MHPSS, symptoms categorized as “extreme” were more prevalent for anxiety (36.38%) and depression (22.55%) (Table 2).

**Table 2:** Distress and functioning categories.

**Category**	**Extreme:**	**Severe:**	**Moderate:**	**Mild:**	**Normal:**
	***n* (%)**	***n* (%)**	***n* (%)**	***n* (%)**	***n* (%)**
**DASS21**
**Depression subscale**
Pre-test (*N* = 3,214)	757 (23.55)	836 (26.01)	1,017 (31.64)	317 (9.86)	287 (8.93)
Post-test (*N* = 1,753)	10 (0.57)	17 (0.97)	138 (7.87)	298 (17.00)	1,290 (73.59)
**Anxiety subscale**
Pre-test (*N* = 3,335)	1,213 (36.37)	683 (20.48)	828 (24.83)	154 (4.62)	457 (13.70)
Post-test (*N* = 1,753)	25 (1.43)	37 (2.11)	191 (10.90)	171 (9.75)	1,329 (75.81)
**Stress subscale**
Pre-test (*N* = 3,335)	91 (2.72)	563 (16.88)	851 (22.52)	642 (19.25)	1,188 (35.62)
Post-test (*N* = 1,753)	0 (0.00)	5 (0.29)	18 (1.03)	60 (3.42)	1,670 (95.27)
**IES-R total score**
Pre-test (*N* = 1,216)	201 (16.53)	727 (59.79)	57 (4.69)	52 (4.28)	179 (14.72)
Post-test (*N* = 544)	0 (0.00)	37 (6.80)	37 (6.80)	134 (24.63)	336 (61.76)
**ICRC functioning scale**
Pre-test (*N* = 2,446)	140 (5.72)	900 (36.79)	997 (40.76)	321 (13.12)	88 (3.60)
Post-test (*N* = 1,308)	68 (5.20)	58 (4.43)	244 (18.65)	269 (20.57)	669 (51.15)

When comparing pre- and post-assessments, DASS21 scores improved among 96.58% of the patients, IES-R scores improved among 92.70% of the patients and scores on the ICRC functioning scale improved among 82.26% of the patients (Table 3).

**Table 3:** Distress and functioning pre- and post-scores.

	**Mean (SD)**	**95% CI**	***p*-value**	**Range**	**Med (IQR)**	**% Who improved**
Pre-DASS (*N* = 3,214)	55.66 (20.62)	54.9; 56.38		2–126	56 (42–70)	
Post-DASS (*N* = 1,753)	17.49 (12.68)	16.89; 18.08		2–88	16 (8–24)	
DASS difference (*N* = 1,669)	−36.71 (19.18)	−37.64; −35.79	<0.0001	−86 to 26	−38 (−48 to −24)	96.58
Pre-IES (*N* = 1,216)	46.90 (18.87)	45.86; 47.94		0–84	50 (40–60)	
Post-IES (*N* = 986)	11.30 (13.20)	10.47; 12.12		0–63	20 (11–27)	
IES difference (*N* = 986)	−34.91	−36.12; −33.69	<0.0001	−74 to 26	−37 (−50 – −20)	92.70
Pre-functioning (*N* = 2,447)	6.20 (0.06)	6.09; 6.31		2–14	6 (4–8)	
Post-functioning (*N* = 1,309)	10.47 (0.10)	10.28; 10.67		2–14	12 (8–14)	
Functioning difference (*N* = 1,302)	4.40 (3.66)	4.20; 4.60	<0.0001	−7–12	4 (2–7)	82.26

*SD, Standard Deviation; CI, Confidence Interval; p-value derived from the Wald test; IQR, interquartile range*.

*Inconsistencies arise due to rounding*.

**Figure 3:**
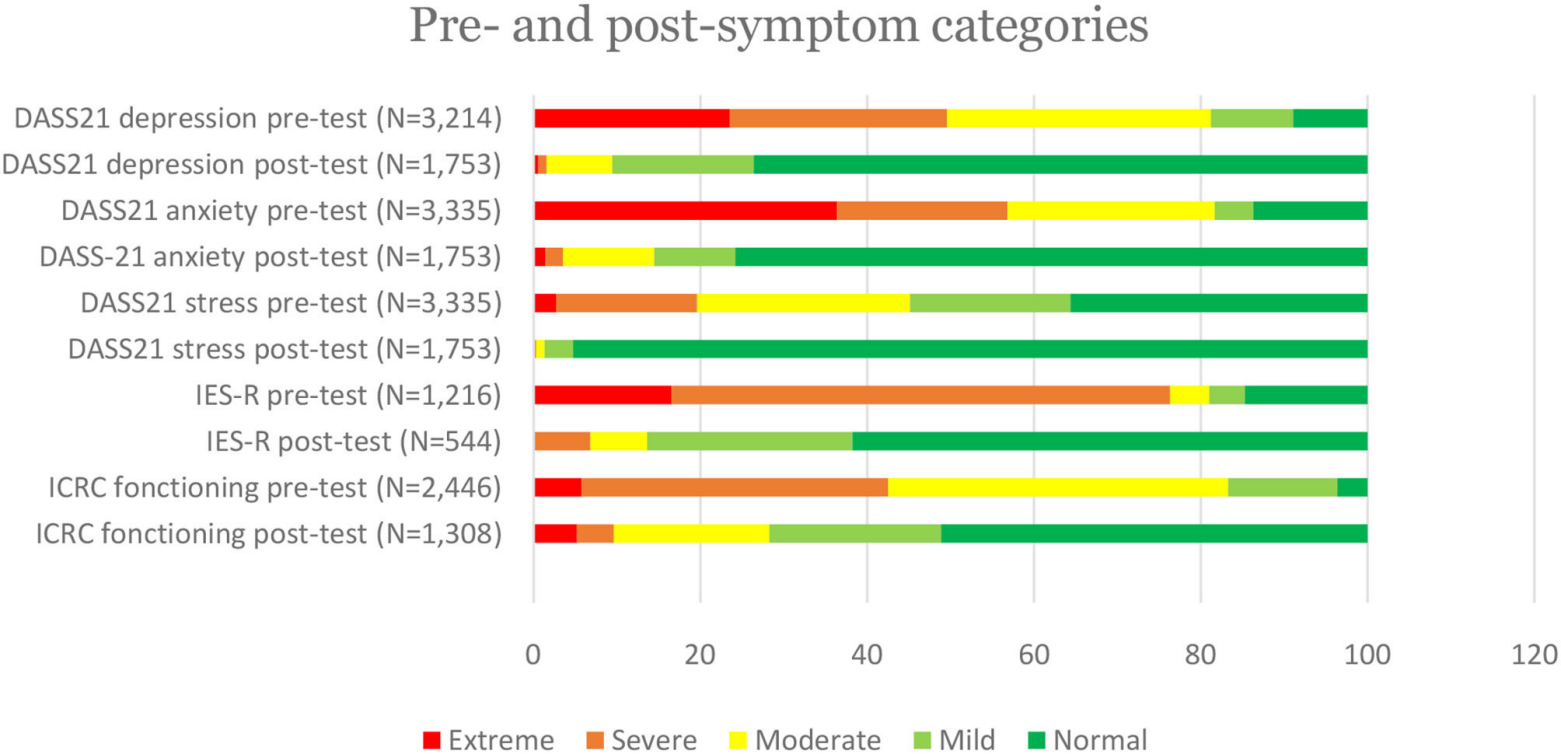
Pre- and post-symptom categories.

The largest reduction in symptoms was seen on the IES-R scale with a 75.9% decrease in mean scores. While very few patients presented “extreme” levels of distress following MHPSS, the majority of patients with “extreme” difficulties in functioning at baseline reported equally “extreme” difficulties at closure (Figure 3).

### Determinants of High Distress and Low Functioning Prior to MHPSS

#### Depression

On the DASS21 scale, high symptoms of depression at intake were associated with particular types of violence; namely forced recruitment (aOR 7.95; *p* = 0.009), witnessing physical violence (aOR 2.54; *p* < 0.0001) and rape (aOR 1.50; *p* < 0.0001). Experiencing discrimination or stigma also increased the likelihood of patients reporting high levels of depression at intake (aOR 2.38; *p* = 0.019), as did destruction or loss of property and/or income (aOR 1.22; *p* = 0.022). Internally displaced beneficiaries were more likely to report high symptoms of depression at intake (aOR 2.25; *p* < 0.0001) as were patients referred by family or friends (aOR 1.40; *p* = 0.023) (Table 4).

**Table 4:** Factors associated with high distress and low functioning at baseline.

**Variables**	**cOR (95%CI)**	***p*-value**	**aOR (95%CI)**	***p*-value**
**Depression (DASS21)**				
**Education level (*****N*** **=** **3,117)**				
Illiterate	Ref	–	Ref	–
Basic	0.95 (0.80; 1.12)	0.538	0.88 (0.71; 1.08)	0.229
Medium	0.85 (0.71; 1.02)	0.080	0.76 (0.61; 0.95)	0.016
High	0.94 (0.37; 2.39)	0.897	0.93 (0.28; 3.14)	0.912
**High anxiety at baseline (*****N*** **=** **3,214)**				
No	Ref	–	Ref	–
Yes	6.13 (5.24; 7.14)	<0.0001	3.77 (3.13; 4.54)	<0.0001
**High stress at baseline (*****N*** **=** **3,214)**				
No	Ref	–	Ref	–
Yes	7.91 (6.74; 9.28)	<0.0001	5.27 (4.39; 6.32)	<0.0001
**Other vulnerability factors (ref** **=** **not reported) (*****N*** **=** **3,214)**				
Destroyed/lost property and/or income	1.35 (1.17; 1.55)	<0.0001	1.22 (1.03; 1.46)	0.022
Experience of discrimination/stigma	2.49 (1.41; 4.41)	0.002	2.38 (1.15; 4.94)	0.019
**Referral pathway (*****N*** **=** **3,212)**				
Self-referred	Ref	–	Ref	–
Referred by family or friends	1.23 (0.98; 1.56)	0.077	1.40 (1.05; 1.87)	0.023
**Internally displaced (*****N*** **=** **3,119)**				
No	Ref	–	Ref	–
Yes	2.29 (1.82; 2.87)	<0.0001	2.25 (1.70; 2.97)	<0.0001
**Type of violence experienced (ref** **=** **not reported) (*****N*** **=** **3,214)**				
Forced recruitment	9.77 (2.27; 42.02)	0.002	7.95 (1.69; 37.47)	0.009
Rape	1.20 (1.04; 1.38)	0.011	1.50 (1.25; 1.80)	<0.0001
Witnessing physical violence	1.86 (1.45; 2.38)	<0.0001	2.54 (1.83; 3.52)	<0.0001
**Anxiety (DASS21)**				
**Alleged perpetrator (*****N*** **=** **3,213)**				
Partner	Ref	–	Ref	–
Family member	1.19 (0.58; 2.46)	0.631	1.51 (0.63; 3.63)	0.356
Known civilian	1.41 (0.73; 2.73)	0.310	2.45 (1.10; 5.48)	0.028
Unknown civilian	1.74 (0.90; 3.38)	0.102	2.76 (1.23; 6.18)	0.014
Military or armed group	2.51 (1.35; 4.69)	0.004	3.51 (1.64; 7.50)	0.001
**High depression at baseline (*****N*** **=** **3,214)**				
No	Ref	–	Ref	–
Yes	6.13 (5.24; 7.17)	<0.0001	3.67 (3.07; 4.39)	<0.0001
**High stress at baseline (*****N*** **=** **3,335)**				
No	Ref	–	Ref	–
Yes	7.11 (6.10; 8.29)	<0.0001	3.87 (3.25; 4.62)	<0.0001
**Other vulnerability factors: lack of social support (*****N*** **=** **3,335)**				
No	Ref	–	Ref	–
Yes	1.77 (1.27; 2.48)	0.001	2.10 (1.39; 3.17)	<0.0001
**Stress (DASS21)**				
**High depression at baseline (*****N*** **=** **3,214)**				
No	Ref	–	Ref	–
Yes	7.91 (6.74; 9.28)	<0.0001	5.23 (4.41; 6.20)	<0.0001
**High anxiety at baseline (*****N*** **=** **3,335)**				
No	Ref	–	Ref	–
Yes	7.11 (6.09; 8.29)	<0.0001	3.90 (3.29; 4.62)	<0.0001
**Type of violence experienced (ref** **=** **not reported) (*****N*** **=** **3,335)**				
Physical violence	1.29 (1.11; 1.49)	0.001	1.49 (1.24; 1.78)	<0.0001
Disappearance/abduction of a loved one	1.68 (0.84; 3.37)	0.145	4.04 (1.10; 14.78)	0.035
**Post-traumatic stress (IES-R)**				
**Age (*****N*** **=** **1,210)**				
0–17	Ref	–	Ref	–
18–24	5.29 (1.14; 24.59)	0.033	5.61 (1.14; 27.50)	0.034
25–34	7.93 (1.79; 35.17)	0.006	8.91 (1.91; 41.59)	0.005
35–44	8.64 (1.95; 38.32)	0.005	9.59 (2.05; 44.81)	0.004
45–81	6.81 (1.54; 30.23)	0.012	9.72 (2.07; 45.56)	0.004
**Gender (*****N*** **=** **1,214)**				
Male	Ref	–	Ref	–
Female	1.86 (1.43; 2.42)	<0.0001	2.07 (1.60; 2.94)	<0.0001
**Education level (*****N*** **=** **1,160)**				
Illiterate	Ref	–	Ref	–
Basic	1.44 (1.11; 1.85)	0.006	1.48 (1.12; 1.94)	0.005
Medium	3.15 (1.95; 5.08)	<0.001	3.38 (2.03; 5.64)	<0.0001
High	1.65 (0.95; 2.89)	0.078	1.78 (0.98; 3.24)	0.060
**Other vulnerability factors (ref** **=** **not reported) (*****N*** **=** **1,216)**				
Lack of social support	2.60 (1.69; 3.99)	<0.0001	2.04 (1.29; 3.22)	0.002
**Type of violence experienced (ref** **=** **not reported) (*****N*** **=** **1,216)**				
Killing of a loved one	2.15 (1.71; 2.71)	<0.0001	1.73 (1.35; 2.22)	<0.0001
Witness to physical violence	1.69 (1.27; 2.25)	<0.0001	1.47 (1.13; 1.91)	0.004
**Functioning (ICRC Africa scale)**				
**High depression at baseline (*****N*** **=** **1,902)**				
No	Ref	–	Ref	–
Yes	1.31 (1.09; 1.57)	0.004	1.28 (1.06; 1.54)	0.010
**Other vulnerability factors (ref** **=** **not reported) (*****N*** **=** **2,447)**				
Destroyed/lost property and/or income	1.77 (1.51; 2.09)	<0.0001	1.80 (1.50; 2.17)	<0.0001
**Type of violence experienced (ref** **=** **not reported) (*****N*** **=** **2,447)**				
Rape	1.05 (0.89; 1.25)	0.540	1.42 (1.16; 1.74)	0.001

*cOR, crude odds ratio; aOR, adjusted odds ratio; p-value from Wald test; DASS21 depression cut-off: 20; DASS21 Anxiety cut-off: 16; DASS21 Stress cut-off: 18; IES-R cut-off: 51; Functioning cut-off: 5*.

#### Anxiety

Predictors of high anxiety at intake included high stress (aOR 3.87; *p* < 0.0001), high depression (aOR 3.67; *p* < 0.0001) and lack of social support (aOR 2.10; *p* < 0.0001). The profile of the alleged perpetrator of the violence also played a role: compared to victims of violence committed by the patients' partners, the likelihood of reporting high anxiety at intake was more than three times higher among victims of violence committed by the military or armed groups (aOR 3.51; *p* = 0.001) and more than twice as high when the alleged perpetrator was an unknown civilian (aOR 2.76; *p* = 0.014) or a known civilian (aOR 2.45; *p* = 0.028).

#### Stress

High stress at baseline correlated with reporting high depression (aOR 5.23; *p* < 0.0001) and high anxiety (aOR 3.90; *p* ≤ 0.0001). In addition, two types of violence were also strong predictors of high distress: disappearance/abduction of a loved one (aOR 4.04; *p* = 0.035) and having experienced physical violence (aOR 1.49; *p* < 0.0001).

#### Post-traumatic Stress

Looking at the IES-R scale, a linear trend (aOR 1.19; *p* = 0.009) was found between increasing likelihood of reporting high symptoms at intake and increasing age. Females were more than twice as likely than males to report high symptoms of post-traumatic stress at intake (aOR 2.07; *p* < 0.0001). The likelihood was higher among patients having commenced primary education (aOR 1.48; *p* = 0.005) and even higher among patients having commenced secondary education (aOR 3.38; *p* < 0.0001). Having experienced particular types of violence increased the likelihood of showing high levels of post-traumatic stress at intake, particularly witnessing the killing of a loved one (aOR 1.73; *p* < 0.0001) and witnessing physical violence in general (aOR 1.47; *p* = 0.004).

#### Functioning

Predictors of low functioning at baseline included high levels of depression (aOR 1.28, *p* = 0.010), having experienced the destruction or loss of property or income (aOR 1.80, *p* ≤ 0.0001) and having been a victim of rape (aOR 1.42; *p* = 0.001).

### Determinants of Improvement Following MHPSS

#### Depression

A linear trend was observed between the length of the treatment and the likelihood of reducing symptoms of depression. No such association was observed between length of treatment and other symptom changes. Patients who had experienced the natural death of a loved one more than two years ago were less likely to report reduced depression at closure (aOR 0.26; *p* < 0.0001) (Table 5).

**Table 5:** Factors associated with improved distress and functioning following MHPSS.

**Variables**	**cOR (95%CI)**	***p*-value**	**aOR (95%CI)**	***p*-value**
**Depression (DASS21)**				
**High depression at baseline (*****N*** **=** **1,669)**				
No	Ref	–	Ref	–
Yes	15.13 (11.92; 19.20)	<0.0001	12.72 (9.57; 16.90)	<0.0001
**Large improvement in symptoms of anxiety (*****N*** **=** **1,669)**				
No	Ref	–	Ref	–
Yes	5.06 (4.11; 6.24)	<0.0001	2.42 (1.82; 3.22)	<0.0001
**Large improvement in symptoms of stress (*****N*** **=** **1,669)**				
No	Ref	–	Ref	–
Yes	6.08 (4.92; 7.52)	<0.0001	2.71 (2.05; 3.60)	<0.0001
**Other vulnerability factors: natural death of loved 1 more than 2 years ago (*****N*** **=** **1,669)**				
No	Ref	–	Ref	–
Yes	0.51 (0.36; 0.74)	<0.0001	0.26 (0.16; 0.43)	<0.0001
**Length of MHPSS: days between pre- and post-assessment (*****N*** **=** **1,552)**				
<8	Ref	–	Ref	–
8–14	1.91 (0.96; 3.78)	0.063	1.29 (0.54; 3.06)	0.561
15–30	4.90 (2.70; 8.89)	<0.0001	1.93 (0.90; 4.12)	0.089
31–60	3.23 (1.77; 5.88)	<0.0001	3.10 (1.46; 6.58)	0.003
61–90	2.99 (1.61; 5.54)	0.001	3.46 (1.59; 7.52)	0.002
91–120	3.52 (1.80; 6.85)	<0.0001	3.66 (1.57; 8.50)	0.003
121–150	3.66 (1.55; 8.65)	0.003	2.77 (0.92; 8.33)	0.071
>150	3.48 (1.48; 8.17)	0.004	2.04 (0.70; 5.97)	0.193
**Anxiety (DASS21)**				
**High anxiety at baseline (*****N*** **=** **1,669)**				
No	Ref	–	Ref	–
Yes	17.28 (13.49; 22.12)	<0.0001	18.73 (14.42; 24.34)	<0.0001
**Education level (*****N*** **=** **1,616)**				
Illiterate	Ref	–	Ref	–
Basic	1.10 (0.86; 1.40)	0.454	0.99 (0.73; 1.36)	0.972
Medium	1.33 (1.03; 1.71)	0.026	1.41 (1.02; 1.94)	0.036
High	1.66 (0.58; 4.74)	0.345	1.22 (0.33; 4.52)	0.771
**Other vulnerability factors: lack of social support (*****N*** **=** **1,669)**				
No	Ref	–	Ref	–
Yes	0.59 (0.38; 0.91)	0.018	0.27 (0.16; 0.45)	<0.0001
**Stress (DASS21)**				
**High stress at baseline (*****N*** **=** **1,669)**				
No	Ref	–	Ref	–
Yes	21.48 (16.46; 28.03)	<0.0001	25.85 (19.47; 34.33)	<0.0001
**Education level (*****N*** **=** **1,616)**				
Illiterate	Ref	–	Ref	–
Basic	1.01 (0.79; 1.30)	0.919	1.14 (0.83; 1.56)	0.429
Medium	1.32 (1.03; 1.70)	0.029	1.65 (1.19; 2.30)	0.003
High	2.47 (0.83; 7.34)	0.104	4.15 (1.05; 16.41)	0.042
**Other vulnerability factors (ref** **=** **not reported) (*****N*** **=** **1,616)**				
Lack of social support	0.88 (0.57; 1.35)	0.548	0.49 (0.28; 0.81)	0.006
Natural death of loved 1 <2 years ago	0.74 (0.54; 1.01)	0.054	0.46 (0.31; 0.68)	<0.0001
**Post-traumatic stress (IES-R)**				
**High post-traumatic stress at baseline (*****N*** **=** **544)**				
No	Ref	–	Ref	–
Yes	2.80 (1.98; 3.97)	<0.0001	1.55 (1.03; 2.33)	0.035
Internally displaced (*N* = 468)				
No	Ref	–	Ref	–
Yes	4.65 (2.18; 9.89)	<0.0001	4.02 (1.84; 8.80)	<0.0001
**Experience of discrimination/stigma/marginalization**				
No	Ref	–	Ref	–
Yes	0.38 (0.10; 1.47)	0.161	0.23 (0.06; 0.92)	0.037
**Occupation**				
Unemployed	Ref	–	Ref	–
Shop or business owner	0.16 (0.06; 0.46)	0.001	0.32 (0.11; 0.95)	0.040
**Functioning (ICRC Africa scale)**				
**Low functioning prior to MHPSS (*****N*** **=** **1,302)**				
No	Ref	–	Ref	–
Yes	1.92 (1.53; 2.40)	<0.0001	1.92 (1.51; 2.44)	<0.0001
Number of children (*N* = 1,171)	1.05 (1.00; 1.10)	0.033	1.05 (1.00; 1.10)	0.042
**Other vulnerability factors: destroyed/lost property and/or income (*****N*** **=** **1,302)**				
No	Ref	–	Ref	–
Yes	0.87 (0.69; 1.08)	0.199	0.90 (0.71; 1.15)	0.044

*cOR, crude odds ratio; aOR, adjusted odds ratio; p-value from Wald test; DASS21 depression cut-off: −14; DASS21 Anxiety cut-off: −12; DASS21 Stress cut-off: −12; IES-R cut-off: −37; Functioning cut-off: 4*.

#### Anxiety

Illiterate patients were less likely to report reduced distress following MHPSS compared to patients with a secondary education level (aOR 1.41, *p* = 0.036). Lack of social support, which was a predictor of high anxiety at baseline, was also a predictor of changes in levels or anxiety following MHPSS. Thus, compared to patients who did not report lacking social support, patients who did report lacking social support were three times less likely to show a large reduction in symptoms of anxiety following MHPSS (aOR 0.27; *p* < 0.0001).

#### Stress

A linear trend was observed between the likelihood of showing a large reduction in symptoms of stress and the patient's education level (OR 1.17; *p* = 0.011). Patients that lacked social support were less than half as likely to show reduced stress at closure (aOR 0.49; *p* = 0.006) as were patients who had experienced the natural death of a loved 1 <2 years ago (aOR 0.46; *p* < 0.0001).

#### Post-traumatic Stress

On the IES-R scale, 92.70% of the patients showed a reduction in symptoms following MHPSS. Experiencing discrimination, stigma and/or marginalization correlated with a smaller or no reduction in symptoms of post-traumatic stress (aOR 0.23; *p* = 0.037).

#### Functioning

A linear trend was found between an increasing number of children and an increasing likelihood of improving daily functioning (aOR 1.05; *p* = 0.042). Also, patients who improved functioning the most were less likely than other patients to have experienced a destruction or loss of property and/or income (aOR 0.90; *p* = 0.044).

#### Individual vs. Group Sessions

No significant differences were found with regard to reduced distress or increased functioning following individual vs. group sessions. However, whereas the average length of individual psychological support was 49 days, the average length of group psychological support was 131 days. Also, the drop-out rate was lower among beneficiaries of group psychological support in that 61.25% completed the sessions with a post-assessment compared to only 43.58% among beneficiaries of individual psychological support.

## Discussion

**Figure 4:**
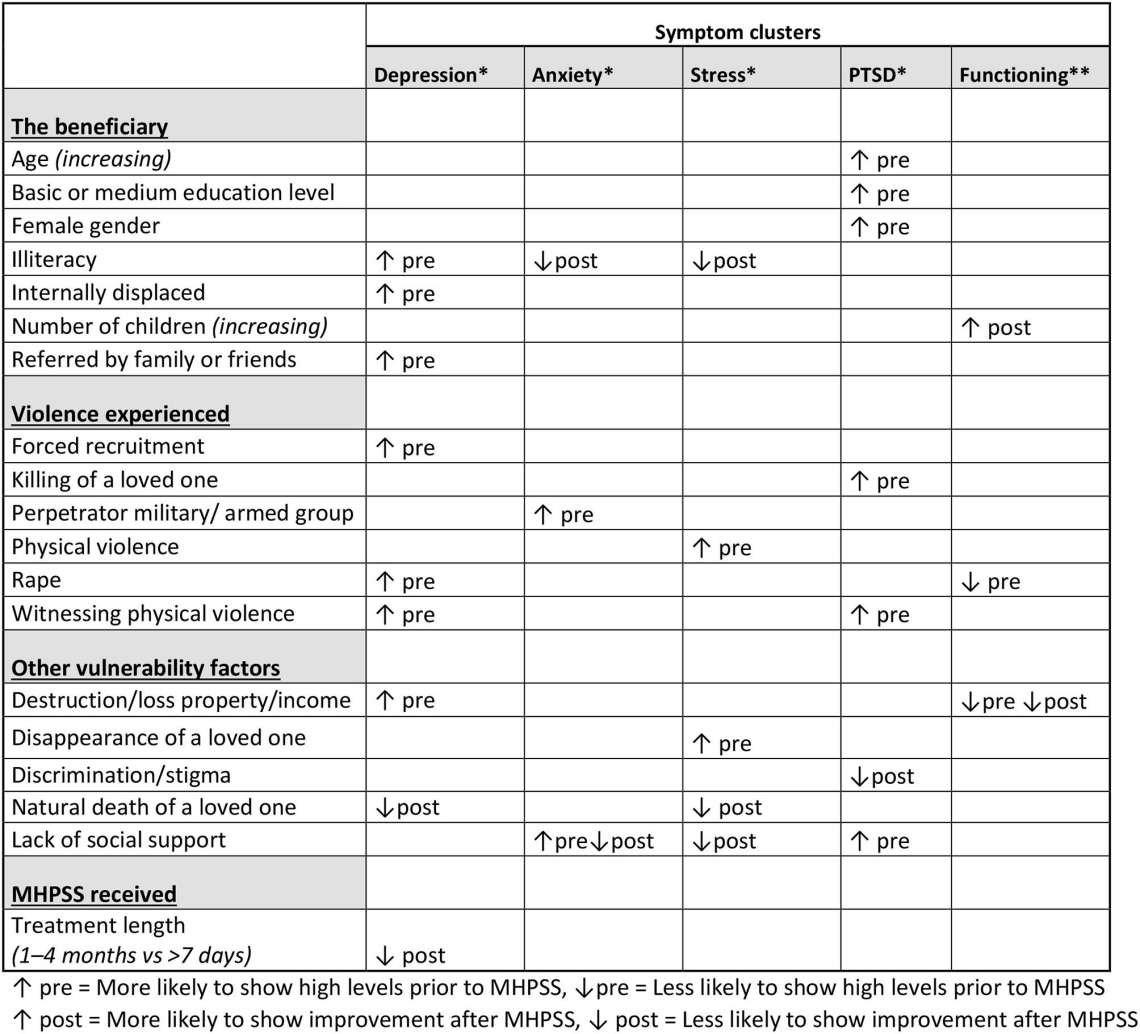
Summary of the main determinants.

The various factors correlating with particular levels of symptoms before and after MHPSS can be divided into four sets of determinants related to the beneficiary, the violence experienced, other vulnerability factors and the psychological support received (Figure 4).

### Determinants Related to the Beneficiary

*Illiteracy* stood out as a strong predictor of high levels of depression. One meta-analysis found a similar association between low socioeconomic status (SES) and an increased likelihood of depression, explained by both causation (low SES increases risk of depression) and selection (depression hinders social mobility). The authors speculated that people of higher socio-economic status (SES) may have more personal resources such as coping mechanisms and self-esteem that can buffer the impact of exposure to violence and stress on depression (13). Testing this hypothesis among beneficiaries of ICRC community-level MHPSS programs would require assessing such personal resources more thoroughly. Following the psychological support, illiterate patients were also less likely to report large reductions in their levels of anxiety and stress. The fact that the better educated beneficiaries improved significantly more on these parameters could indicate that the psychological support, in its current form, contributes to favoring the better educated patients. Thus, it would seem relevant to review the current approach to ensure that illiterate patients receive appropriate psychological support to address anxiety and stress in particular.

*Internal displacement* also correlated with increased likelihood of high depression at baseline. A recent meta-analysis confirmed a link between internal displacement and depression rates of up to 80% (14). The fact that internal displacement did not influence the outcome of the psychological support suggests that the counseling in its current form adequately addresses the needs of this sub-group of beneficiaries.

The referral pathway played a role in that beneficiaries *referred by family members or friends* were more likely to present high symptoms of depression at baseline. This suggests that people with the most severe levels of depression are less likely to proactively seek care by themselves and that identifying these beneficiaries through their family and friends is essential to ensuring that they gain access to psychological support. This finding should be taken into consideration when setting down strategies for awareness-raising. Such activities should target not only potential beneficiaries, but also members of the community who may not need MHPSS services themselves but who can help to identify vulnerable people in their network in need of this type of support.

High levels of post-traumatic stress were associated with *increasing age, primary and secondary education level* and *female gender*. While the first two are not consistent with findings of other studies (13, 15), the correlation between female gender and increased likelihood of post-traumatic stress disorder (PTSD), including prolonged duration of PTSD, has been shown both in Western (16) and Africa (17) populations.

Unexpectedly, the *more children* the beneficiary had, the more likely he or she was to show increased functioning following psychological support. The authors are not aware of any studies documenting such an association. It is likely that having more children to take care of pushes the beneficiary out of ruminations and back to doing what is necessary, i.e., functioning. Also, one of the elements of the psychological support is to mobilize one's network (including children) to increase practical and emotional support. This may have helped some beneficiaries to more easily carry out everyday tasks.

### Determinants Related to the Violence Experienced

High symptoms of depression correlated with having experienced *forced recruitment, rape* or w*itnessing physical violence*. A study of former child soldiers in Uganda found depression to be the most dominant mental health disorder with a prevalence rate of 36% (18). A systematic review found forced recruitment to increase the risk of both depression and PTSD, despite variations in prevalence rates depending on the methodology used in the various studies (19).

Consistent with the association between rape and increased likelihood of high symptoms of depression, a systematic review (20) found prevalence rates of depression of up to 76% among victims of sexual violence in conflict settings. The fact that rape also correlated with low functioning prior to receiving psychological support points to the very tangible consequences that this grave form of sexual violence has on the life of the victims.

Furthermore, consistent with the association between witnessing of physical violence and increased likelihood of high symptoms of depression, a study from a non-humanitarian setting found that witnessing community violence, without ever being a direct victim of physical violence, more than doubled the likelihood of depression (21).

The profile of the perpetrator as weapon bearer vs. civilian matters not only in terms of the ICRC mandate (22), but also in terms of predicting high levels of anxiety among victims of violence. Indeed, addressing the psychological needs of victims of the violence committed by a member of the military or an armed group with, for example a generalized fear of men in uniforms, differs from addressing the psychological needs of, for example, a victim of domestic violence.

These types of violence correlated with the levels of certain symptoms prior to—but not following—psychological support. This indicates that while counselors should be attentive to certain types of violence causing particular psychological needs, by and large the psychological support in its current form does not overlook the needs of beneficiaries having experienced particular types of violence.

### Determinants Related to Other Vulnerability Factors

*Disappearance* of a loved one predicted high levels of stress prior to psychological support. The link between having a missing relative and experiencing psychological distress has been documented in a previous study of ICRC MHPSS programs (23). Likewise, social *stigma* was a predictor of depression. One study found that stigma has been identified as a mediator between sexual violence and depression in the DRC (24).

Three predictors of high distress and low functioning both before and after receiving psychological support stood out repeatedly (see Figure 2): *lack of social support, grief* and *acute financial needs* resulting from destruction or loss of property or income. These findings suggest that beneficiaries presenting these additional vulnerability factors benefit less from the psychological support that they receive. Consequently, when relevant, the counseling should focus on expanding the network of beneficiaries with limited or no social support, and counselors should be attentive to beneficiaries experiencing grief even when caused by factors unrelated to the armed conflict. Also, for beneficiaries with acute financial needs resulting from exposure to violence, psychological support alone does not suffice. For this sub-group of beneficiaries, MHPSS outcomes such as reduced psychological distress cannot be achieved unless financial needs are addressed in parallel with the psychological support.

### Determinants Related to the MHPSS Received

The only determinant of improvement that was related to the MHPSS received was the *length* of the psychological support, which can also be explained simply by the passage of time. No significant difference in improvement was found between beneficiaries receiving individual and group psychological support *per se*, although group support tended to last almost three times longer than individual support. Thus, increasing the length of the psychological support, particularly for the individual support, should be prioritized to ensure that beneficiaries profit as much as possible. This is particularly challenging in conflict settings characterized by limited access and internal displacement.

However, if the aim is truly to make a difference by reducing the psychological distress and increasing the daily functioning of victims of violence, then the barriers to longer-term psychological support must be further examined and addressed. Even from a strict cost-benefit perspective, having already invested in having specialized MHPSS teams on the ground, organizing training sessions and supervisions, paying incentives etc., it would make sense to try to understand and address the barriers to longer-term treatment adherence more thoroughly.

### Strengths and Limitations

This study was made possible by the extensive monitoring system of ICRC MHPSS programs. The real-life settings, the uniqueness of the data and the large number of beneficiaries involved constitute major strengths of this study. Furthermore, the quality of the data derived from standardized psychometric tools and following each individual patient before and after MHPSS can be considered as important attributes.

The main limitation of this study is the absence of a control group. It is not possible to state whether the changes in distress levels were due to the intervention or other reasons, even if the experience of violence preceded the MHPSS intervention. Thus, the study does not claim to have identified causal relationships, only associations.

Another limitation is the fact that all the data used in the study stems from information obtained from the patient him or herself; this may have introduced information bias and possible misclassification. Finally, despite the various variables used to construct the regression models, residual confounding cannot be ruled out.

## Conclusion

Supplemented by MHPSS projects integrated into primary health care, hospitals, physical rehabilitation centers and other structures, community-level projects remain a pertinent entry point for reaching the broader civilian population affected by armed conflict and other violence.

The main operational recommendations deriving from this study can be summarized as follows:

- Using qualitative methods, explore the link between illiteracy and reduced likelihood of improvement in anxiety and stress following MHPSS.
- Where relevant, ensure greater adaptation of the psychological support to beneficiaries with a low education level.
- Address *grief* by reinforcing the technical capacity of the counselors on this topic.
- Address *lack of social support* more proactively by enhancing, when relevant, the beneficiary's support network.
- Ensure that acute financial needs resulting from violence are addressed alongside the psychological needs.
- Examine and address barriers to adherence to treatment, particularly for individual psychological support, to increase the number of follow-up sessions per patient. Explore the pertinence of reimbursing transportation costs and/or introducing more home visits.
- Monitor the content of the psychological support, i.e., techniques used and themes discussed, to be able to pinpoint and reinforce the specific aspects that correlate with improvement.
- Monitor and analyze to what extent interventions at lower levels of the pyramid prevent or otherwise influence the needs at the psychological support layer.
- Monitor and analyze to what extent referrals to other service providers influence psychological and other outcomes.
- Conduct a longitudinal study to assess the longer-term levels of distress and functioning following MHPSS.

## Data Availability Statement

The original contributions presented in the study are included in the article/Supplementary Material, further inquiries can be directed to the corresponding author.

## Ethics Statement

The studies involving human participants were reviewed and approved by the Medical Ethics Committee of the Free University of Brussels (VUB) in Brussels, Belgium (B.U.N. 143201942389). The data were not initially collected for research purposes, but as part of the routine monitoring of the ICRC MHPSS sub-unit. Written informed consent from the participants' legal guardian/next of kin was not required to participate in this study in accordance with the national legislation and the institutional requirements.

## Author Contributions

RR conceived and designed the analysis. IA compiled the data, performed the analysis, and wrote the paper. IA and RR contributed data or analysis tools. RR and IH commented and validated the final draft. All authors contributed to the article and approved the submitted version.

## Funding

This study was carried out within the framework of the International Doctoral Programme in Global Health, Humanitarian Aid and Disaster Management offered jointly by the Center for Research and Training in Disaster Medicine (CRIMEDIM) at the University Piémonte Orientale (UPO) and the Research Group on Emergency and Disaster Medicine (ReGEDiM) at the Vrije Universiteit Brussel (VUB).

## Author Disclaimer

The views expressed are those of the authors and do not necessarily represent the views, policies or decisions of their employers.

## Conflict of Interest

The authors declare that the research was conducted in the absence of any commercial or financial relationships that could be construed as a potential conflict of interest.

## Publisher's Note

All claims expressed in this article are solely those of the authors and do not necessarily represent those of their affiliated organizations, or those of the publisher, the editors and the reviewers. Any product that may be evaluated in this article, or claim that may be made by its manufacturer, is not guaranteed or endorsed by the publisher.

## Footnotes

^1^https://rcrcconference.org/about/33rd-international-conference/ (accessed May 23, 2021).
